# Anger while driving in Mexico City

**DOI:** 10.1371/journal.pone.0223048

**Published:** 2019-09-30

**Authors:** Ana María Hernández-Hernández, Jesús M. Siqueiros-García, Eduardo Robles-Belmont, Carlos Gershenson

**Affiliations:** 1 Instituto de Investigaciones en Matemáticas Aplicadas y en Sistemas, Universidad Nacional Autónoma de México, Ciudad de México, Distrito Federal, México; 2 Centro de Ciencias de la Complejidad, Universidad Nacional Autónoma de México, Ciudad de México, Distrito Federal, México; 3 ITMO University, St. Petersburg, Russia Federation; Universitat de Valencia, SPAIN

## Abstract

This study aims to analyze the level of anger developed by drivers in Mexico City and also understand the behavior that those drivers use to express that anger, using four different survey methods. The first focuses on personal information, the second Driving Anger Expression Inventory (DAX), the third refers to a shorten version of Driving Anger Scale (DAS) and the fourth being the Dula Dangerous Driving Index (DDDI). These have previously been applied and validated in several different countries. The questionnaires were filled out online by 626 drivers. Using the data collected through the online platform, it was possible to identify the kind of reactions volunteers displayed while driving. Also, it was possible to identify that people in Mexico City developed anger depending on their driving area. Our analyses shows that in the Adaptive/Constructive Expression subscale, males and females show a significant difference in their mean score, with women express their anger in a more constructive way than males.

## Introduction

In the last three decades, traffic and human mobility in big cities have been topics of increasing interest for the social [1–4] and computer science [5–8]. These topics have been researched to understand the way that people move in the city through street networks [9–12] or public transportation networks [13–15], but also to understand people’s behavior as they commute [16, 17]. In this regard, one of the most significant efforts is the study and understanding of “driving anger” [18]. In 1994, Deffenbacher et al. created an instrument called Driving Anger Scale (DAS) which was specifically designed to study this emotion [18]. Ever since, others have applied this instrument in different countries [19–23]. For example in Japan, the average score for the complete Driving Anger Scale (33 items) was 69.2, while in the United States the average score was 109.0. Researchers found that Japanese drivers are more sensitive and feel more anger when hostile gestures are displayed but overall a lower feelings of anger about discourtesy compared to western countries [24]. Another study found that taxi drivers in New Zealand feel angrier when someone runs a red light or when someone backs out right in front of them without looking [25]. The study of driving anger (as an emotion and as the behavior attached to it) is essential as cities become denser, and interactions with others become more frequent. Under these circumstances, driver’s emotions can affect the drivers’ behavior, which can have effects on the global patterns of traffic [8] and can generate potential risks to others drivers, bikers or pedestrians [26, 27].

Over the years, other instruments have been created for understanding emotions and behavior displayed by driver’s [18, 28–36]. For example, in 2002 Deffenbacher et al. created the Driving Anger Expression Inventory (DAX) and in 2003 Dula et al., introduced the Dula Dangerous Driving Index (DDDI). The former instrument was desgined to study the behavior of drivers and their expression of anger [32, 37, 38] which later aimed to understand anger as an emotion and also show how anger can be expressed [30, 34]. Such instruments have been widely used and have made it possible to compare similarities and differences between the responses of drivers from different cities and countries [19, 20, 22, 23, 30, 34, 36, 39–41].

Mexico has been no exception in studying these topics [41–45]. For example, Dorantes-Argandar created and validated an inventory instrument to study the aggressive driving behaviours in traffic by taking three groups of data [41]. The size of the sample for each case was 247, 444 an 1763 participants recruited in different populations of the Mexican State of Morelos. In another study Alcazár-Olán et al., [43], did a validation of the DAX survey (49 items). In this study, the data was acquired from 987 Mexican students from a private university with an average age of 21.24 years. In this case, the validation of the whole DAX instrument was not satisfactory and it was later reduced to 33 items.

DAS, DAX, DDDI and other related instruments had also been used for the analysis of drivers response according to gender, age and work conditions [22, 23, 29, 34, 46–58]. For example, Sullman found that females display a higher level of anger due to risky driving on the 33-items DAS survey [48]. In another experiment, Sullman, Stephens and Yong found that younger drivers become angrier because of discourtesy, traffic obstruction, hostile gestures, slow driving and police presence on the 33-items DAS survey [23]. Also, Escanés et al., analysis indicates that for two sub-scales (progress impeded and hostile gestures) on the short DAS survey, females show a higher mean score than males [59].

For the DAX survey, several works have been performed studying the differences in the expression of anger between males and females [49–53]. For example, Eşiyok, Yasak, and Korkusuz found that males between 21 and 30 years report a higher expression of anger through physical means compared to other age groups [51]. In an analysis by age, Jovanović, Lipovac and Stanojević found that older drivers report to express less anger while driving [50]. Finally, for the DDDI survey, there are some differences between males and females. Dula and Ballard found that males obtained a higher score in aggressive and risky driving [30].

The objective of this work is to study if differences exist between the level of anger and behavior of different individuals accordingly with their characteristics. If these differences exist, the situations and behaviors can be included in agent-based modeling. The article is structured in the following manner. First we present materials and methods used. We explain how we designed and ran an online survey based on the DAX, DAS and DDDI instruments. Second, we introduce the results of five different analyses, one based on gender, a second analysis based on the age of the participants, the third focused on driving time, a fourth analysis was made taking into account whether people drive more because of work requirements, and a fifth analysis was made based on the distance from home to the work place. Finally we have a discussion and conclusions.

## Materials and methods

For this research, we designed an online platform to collect data from volunteers. To begin with, participants needed to register themselves and create a user ID to answer the four surveys. Before the completion of the registration process, volunteers had to read and agree to consent form provided before they could participate. Finally, all information collected was anonymous. The research protocol was properly evaluated and approved by the Academic Council of the Research Institute in Applied Mathematics and Systems (Instituto de Investigaciones en Matemáticas Aplicadas y en Sistemas IIMAS-UNAM). Data were collected accordingly, once it was approved. This research do not imply any risk for the participants. None of the surveys questions asked for information which could harm the participant in any way.

### Surveys

The first questionnaire focused on personal and geographic information such as gender, age, working place, living place, and usual hours of driving (for more detail see Supporting Information S1 File). The second survey was the Driving Anger Expression Inventory (DAX) which addresses the expression of anger [32, 38]. The version used in this research has 49 questions organized under four sub-scales. The first sub-scale is the Verbally Aggressive Expression (VAE) and has twelve questions. The VAE was originally designed to obtain information about the verbal expression of anger towards another driver when it is believed that someone was wronged. The second sub-scale is called the Physically Aggressive Expression (PAE), and it is composed of eleven items. This sub-scale is related with the obscene gestures or physical confrontation with other drivers. The third sub-scale is called Using the Vehicle for Aggressive Expression (UVAE), and it sums up to eleven questions. The last sub-scale is called Adaptive/Constructive Expression (ACE). It includes fifteen items and it is related to the strategies adopted by drivers to calm down when they feel anger while driving. DAX is a multiple choice questionnaire for which there are four possible responses with a value ranging from 1 to 4: 1—Almost never, 2—Sometimes, 3—Often, and 4—Almost always [32, 38].

The third survey is a modified version of the Driving Anger Scale (DAS) [18, 29] which is useful to know the level of anger driver’s experience. This survey is divided into four sub-scales. The first subscale has six items, and it is called the Traffic Obstruction (TO). It is associated with driver’s reactions to people or things that obstructed their way. The second sub-scale measures the response to Discourtesy (Dis), and it has five questions. This sub-scale is related to other driver’s behaviour that produces an uncomfortable feeling. The third sub-scale, Illegal Driving (ID), has six items and is associated with other drivers breaking rules. The last sub-scale, Slow Driving (SL), has three items. The options to answer any question from the DAS survey range from 1 to 5; 1—None at all, 2—A little, 3—Some, 4—Much, and 5—Very much [18, 29].

The final instrument is the Dula Dangerous Driving Index (DDDI) [30, 34] which is a survey that focuses on both, emotions and behaviours. This survey has thirty-one questions divided into four sub-scales. The first sub-scale is related to the Aggressive Driving (AD) and has seven items. This sub-scale is associated to the revenge feeling a driver displays when another driver does something wrong. Negative Emotions while Driving (NE), is the second sub-scale and is composed of nine questions related to the development of anger and negative feelings against another driver. The next sub-scale is Risky Driving (RD) (twelve items), and it is associated with the decisions taken against the security of other drivers if they seem to be doing something wrong. The last is connected to questions omitted (O) from the previous three sub-scales and has three items. for the DDDI questionarie, the numerical scale is given by multiple options which take values from 1 to 5; 1—Never, 2—Rarely, 3—Sometimes, 4—Often, and 5—always [30, 34]. Once the questions have been answered a score for each sub-scale is obtained by adding the values of each item that belong to that sub-scale and then the result is divided by the number of questions on the sub-scale. The same procedure is followed to obtain the total score of the survey.

The web platform was available at the Mathematical Modeling of Social Systems department server (http://mmss.iimas.unam.mx/IIMAS-ira-por-manejo/inicio.html) and was publicized through different methods like radio-UNAM, TV-UNAM, and through social platforms such as Facebook and Twitter. The diffusion of the surveys was made to get participants into the study and obtain the necessary data. Once the volunteers filled out the questionnaires, the data analysis began.

### Participants and data collection

Mexico City is a good example of a Megalopolis in which driving and traffic is an everyday problem [60, 61]. Mexico City has an area of 1,495 *Km*^2^, has a population of almost 9 million people and a density of 5,966 *people*/*Km*^2^, However the whole metropolitan area reaches 20.4 million people [62]. People move through the city using different commuting services such as the subway system, buses and troellybuses. On 2001, the metropolitan area included around 3.3 millions vehicles (i.e. cars, buses, motorcycles, etc.) from which 2,341,731 are cars owned for personal transportation [63]. In 2017, a total of 5,471,904 vehicles were registered in Mexico City, 5,008,454 were cars, 32,245 were buses, 83,354 were cargo vehicles and 347,851 were motorcycles [64]. Taking the total number of vehicules registered in Mexico City for 2017 as the universe, the sample size for a confidence level of 95% and a margin error of 5% is 385 drivers [65]. However, as our interest was to analyze the data by gender, age, approximate driving time, driving associated with work, travel distance and housing, the total size was 626 volunteers. In this study, any person who drives, lives in Mexico City and is over 18 years old could participate. There was no other restriction to participate. The data was collected from May to June 2018 through the web platform designed for this purpose. A total of 1533 people accessed the platform. From the total, 626 were taken into account for the study. The other 907 participants were not included because they did not fill all the questionnaires or did not live in Mexico City.

### Data analyses

**Fig 1:**
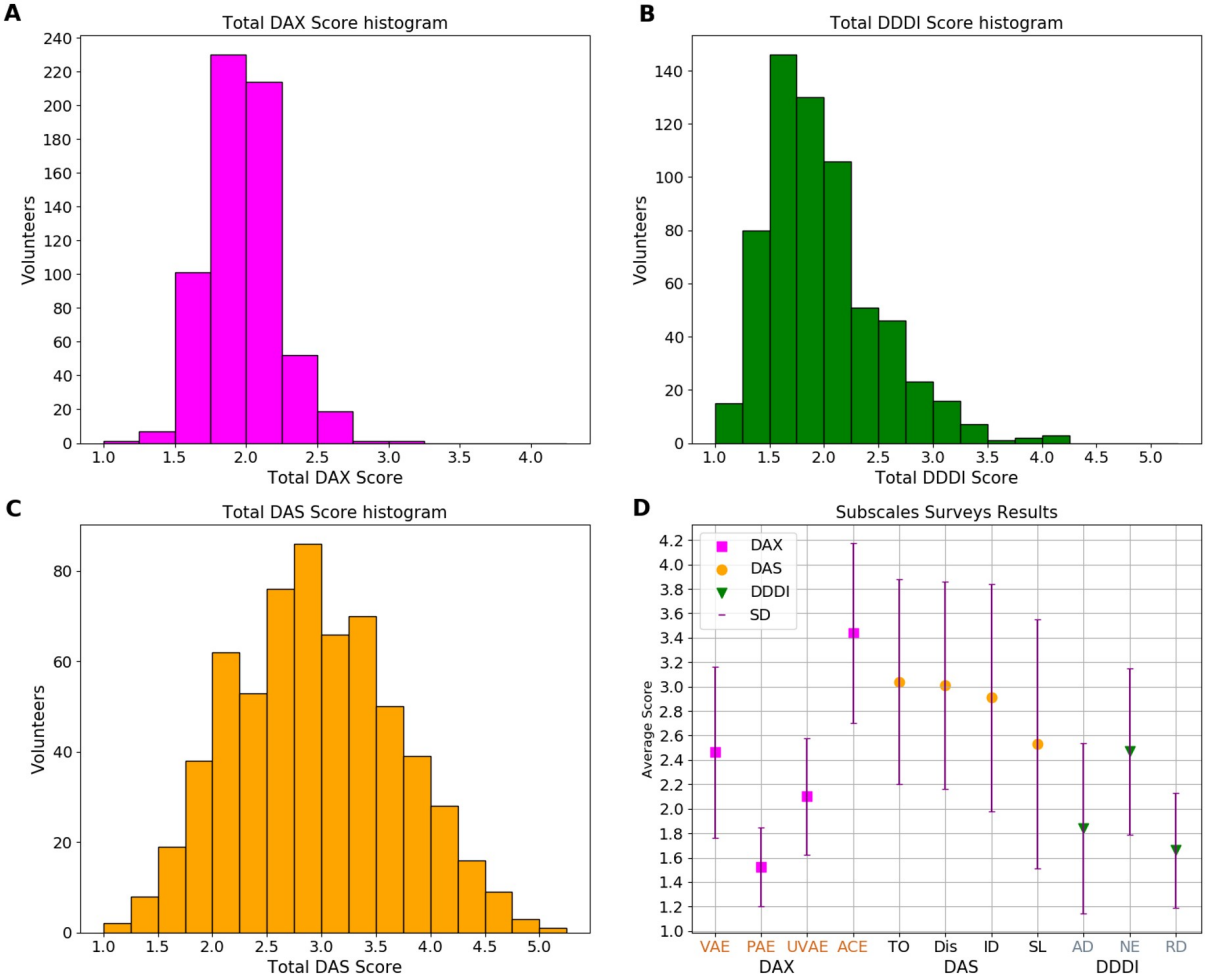
DAX, DAS and DDDI total scores for the sample of 626 drivers in Mexico City. A. Histogram associated to DAX instrument. B. Histogram from scores obtained for DDDI survey. C. Histogram from scores obtained for DAS questionarie. D. Average score and standard desviation for sub-scales from each instrument. The DAX score obtained in each case was multiplied by 1.25 to have 5 as a maximum value.

**Fig 2:**
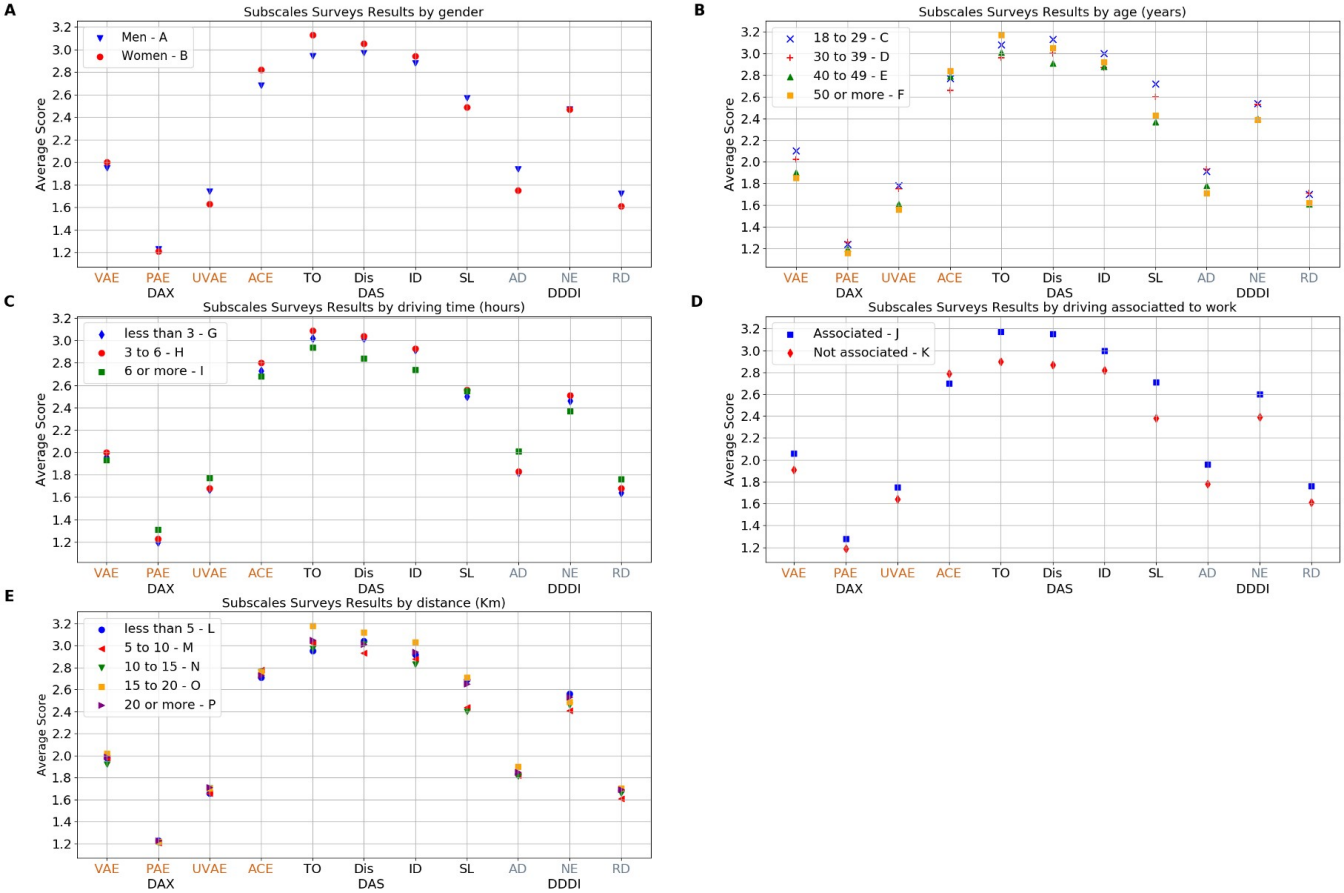
DAX, DAS and DDDI total scores by gender, age, driving time and origin-destination distance. A. Average score for males and females for each subscale. B. Average score for each subscale by age group. C. Average score for each subscale by driving time. D. Average score for each subscale by driving associated to work. E. Average score for each subscale by origin-destination distance.

In order to characterize the sample population we used the personal and geographical information obtained from the first questionnaire. Once the analysis from the first survey was performed, data collected through the DAX, DAS and DDDI surveys were analyzed for the whole sample to obtain a general result (Fig 1, Table 1). Then, the entire sample was divided into groups for a series of analyses. The first analysis by groups was made for gender (Table 2). The second analysis by groups was elaborated based on the age of the participants (Table 3). The third focused on driving time (Table 4). The fourth took into account whether people drive more because of work requirements (Table 5) and the fifth was made taking into account the distance from home to the work place (Table 6, for all groups see Fig 2). Those distances from origin to destination were calculated using Python’s OSMnx and NewtorkX libraries. OSMnx has a MIT license and NetwokX has a BSD license [66–69].

**Table 1:** Survey results for Mexico City. Global DAX, DAS and DDDI results for Mexico City (N = 626).

Survey and Subscale	Cronbach’s *α*	MEAN (SD)
**Survey 2**		
Driving Anger Expression Inventory—Total Score **(DAX)**	0.80	1.97 (0.25)
*Subscales*		
Verbally Aggressive Expression **(VAE)**	0.87	1.97 (0.56)
Physically Aggressive Expression **(PAE)**	0.71	1.22 (0.26)
Use of Vehicle for Aggressive Expression **(UVAE)**	0.77	1.60 (0.44)
Adaptive/Constructive Expression **(ACE)**	0.88	2.75 (0.59)
**Survey 3**		
Driving Anger Scale—Total Score **(DAS)**	0.92	2.92 (0.75)
*Subscales*		
Traffic Obstruction **(TO)**	0.79	3.04 (0.84)
Discourtesy **(Dis)**	0.79	3.01 (0.85)
Illegal Driving **(ID)**	0.85	2.91 (0.93)
Slow Driving **(SL)**	0.80	2.53 (1.02)
**Survey 4**		
Dula Dangerous Driving Index—Total Score **(DDDI)**	0.91	1.96 (0.51)
*Subscales*		
Aggressive Driving **(AD)**	0.82	1.84 (0.70)
Negative Emotions **(NE)**	0.85	2.47 (0.68)
Risky Driving **(RD)**	0.77	1.66 (0.47)

**Table 2:** Survey results by gender. DAX, DAS and DDDI results for Mexico City (N = 626) analyzed by gender. In this sample, there are 302 males and 324 females. Mean(Standard Deviation). ^•^ indicates the sub-scales in which it was visible a significant difference p≤ 0.05.

Survey subscale	Gender—Group		t	p
	Male—A	Female—B		
**DAX**	1.95(0.27)	1.98(0.23)	1.24	0.21
VAE	1.95(0.57)	2.00(0.55)	1.09	0.27
PAE	1.23(0.28)	1.21(0.24)	0.90	0.36
UVAE	1.67(0.48)	1.55(0.38)	3.62	0.0003^•^
ACE	2.68(0.61)	2.82(0.56)	2.90	0.003^•^
**DAS**	2.87(0.76)	2.95(0.74)	1.37	0.17
TO	2.94(0.82)	3.13(0.82)	2.70	0.007^•^
Dis	2.97(0.86)	3.05(0.84)	1.24	0.21
ID	2.88(0.92)	2.94(0.93)	0.74	0.45
SL	2.57(1.02)	2.49(1.01)	0.95	0.33
**DDDI**	2.03(0.54)	1.92(0.44)	2.41	0.01^•^
AD	1.94(0.78)	1.75(0.59)	3.52	0.0004^•^
NE	2.47(0.73)	2.47(0.63)	0.08	0.93
RD	1.72(0.51)	1.61(0.42)	3.17	0.001^•^

**Table 3:** Survey results by age. DAX, DAS and DDDI results for Mexico City (N = 626) analyzed by age. In this case, 129 volunteers are 18 to 29 years, 198 are 30 to 39 years, 184 are 40 to 49 and 115 are 50 years or older. Mean(Standard Deviation). The last column of the table shows the groups which have a significant difference between based on the Tukey post hoc test.

Survey subscale	Anova		Ages range—Group				Compared groups with significant differences *
	F-value	p-value	18 to 29—C	30 to 39—D	40 to 49—E	> 50—F	
**DAX**	4.96	0.002	2.03(0.23)	1.98(0.27)	1.94(0.24)	1.92(0.19)	(C-E) (C-F)
VAE	5.90	0.001	2.10(0.56)	2.02(0.59)	1.90(0.56)	1.85(0.46)	(C-E),(C-F),(D-F)
PAE	3.90	0.009	1.24(0.25)	1.26(0.31)	1.20(0.24)	1.16(0.18)	(D-F)
UVAE	11.21	0.0001	1.70(0.43)	1.69(0.48)	1.54(0.39)	1.46(0.34)	(C-E),(C-F),(D-E),(D-F)
ACE	2.96	0.03	2.77(0.53)	2.66(0.64)	2.79(0.59)	2.84(0.53)	(D-F)
**DAS**	1.43	0.23	3.01(0.70)	2.89(0.74)	2.85(0.79)	2.87(0.86)	None
TO	1.78	0.14	3.08(0.81)	2.96(0.91)	3.01(0.91)	3.17(0.78)	None
Dis	1.68	0.16	3.13(0.86)	3.00(0.84)	2.91(0.86)	3.05(0.84)	None
ID	0.65	0.57	3.00(0.89)	2.87(0.92)	2.88(0.97)	2.92(0.92)	None
SL	4.01	0.008	2.72(1.08)	2.60(1.03)	2.37(0.98)	2.43(0.92)	(C-E)
**DDDI**	3.35	0.01	2.02(0.49)	2.03(0.56)	1.90(0.49)	1.88(0.43)	(C-E),(C-F)
AD	3.40	0.01	1.91(0.70)	1.93(0.77)	1.78(0.65)	1.71(0.59)	(D-F)
NE	2.17	0.09	2.54(0.61)	2.53(0.75)	2.40(0.67)	2.39(0.60)	None
RD	2.25	0.08	1.70(0.51)	1.71(0.49)	1.61(0.44)	1.62(0.41)	None

* p ≤ 0.05

**Table 4:** Survey results by approximate driving time. DAX, DAS and DDDI results for Mexico City (N = 626) drivers taking driving time into account. From the sample, 328 volunteers drive from 0.5 to 3 hours per day, 243 drive 3 to 6 hours per day and 55 drive 6 or more hours. Mean(Standard Deviation).

Survey subscale	Anova		Driving Time—Group		
	F-value	p-value	< 3 hours—G	3 to 6 hours—H	> 6 hours—I
**DAX**	2.29	0.10	1.95(0.23)	1.99(0.24)	1.98(0.30)
VAE	0.67	0.51	1.96(0.56)	2.00(0.55)	1.93(0.61)
PAE	5.52	0.004	1.20(0.24)*	1.23(0.25)	1.31(0.37)*
UVAE	2.13	0.11	1.59(0.43)	1.60(0.38)	1.72(0.59)
ACE	1.49	0.22	2.73(0.57)	2.80(0.56)	2.68(0.76)
**DAS**	0.94	0.39	2.91(0.70)	2.95(0.78)	2.80(0.88)
TO	0.86	0.42	3.02(0.81)	3.09(0.86)	2.94(0.97)
Dis	1.32	0.26	3.02(0.81)	3.04(0.88)	2.84(0.94)
ID	0.98	0.37	2.92(0.88)	2.93(0.96)	2.74(1.04)
SL	0.27	0.75	2.50(0.97)	2.56(1.04)	2.55(1.16)
**DDDI**	0.67	0.51	1.94(0.48)	1.98(0.49)	2.01(0.68)
AD	1.81	0.16	1.82(0.65)	1.83(0.66)	2.01(1.02)
NE	1.04	0.35	2.46(0.65)	2.51(0.69)	2.37(0.75)
RD	1.70	0.18	1.64(0.45)	1.68(0.47)	1.76(0.57)

* significant difference between the groups with p ≤ 0.05 in post hoc Tukey test.

**Table 5:** Survey results by driving associated to work. DAX, DAS and DDDI results for Mexico City (N = 626) drivers taking into account if they drive because of work. From the sample, 204 mention that they drive because of work and 422 mention that they drive not because of work. Mean(Standard Deviation). ^•^ indicates the sub-scales in which it was visible a significant difference.

Survey subscale	Driving associated to work—Group		t	p
	Associated—J	Not associated—K		
**DAX**	2.00(0.26)	1.95(0.23)	2,31	0.01^•^
VAE	2.06(0.58)	1.91(0.55)	2.86	0.004^•^
PAE	1.28(0.30)	1.19(0.24)	3.61	0.0003^•^
UVAE	1.68(0.48)	1.57(0.40)	2.92	0.003^•^
ACE	2.70(0.66)	2.79(0.56)	1.50	0.1
**DAS**	3.04(0.82)	2.79(0.70)	2.88	0.004^•^
TO	3.17(0.90)	2.97(0.82)	2.63	0.008^•^
Dis	3.15(0.90)	2.87(0.82)	2.79	0.005^•^
ID	3.00(1.00)	2.82(0.87)	1.61	0.1
SL	2.71(1.06)	2.38(0.96)	3.16	0.001^•^
**DDDI**	2.08(0.58)	1.91(0.45)	3.73	0.0002^•^
AD	1.96(0.81)	1.78(0.64)	2.83	0.004^•^
NE	2.60(0.76)	2.39(0.67)	3.41	0.0007^•^
RD	1.76(0.52)	1.61(0.45)	3.64	0.0003^•^

**Table 6:** Survey results by living and working place distance. DAX, DAS and DDDI results for Mexico City (N = 517). From this reduced sample, 86 surveyees drive less than 5 Km from home to work, 171 travel from 5 to 10 Km, 129 drive from 10 to 15 Km, 63 from 15 to 20 Km and 68 travel 20 or more Kilometers. Mean(Standard Deviation).

Survey subscale	Anova		Distance from home to work—Group				
	F-value	p-value	< 5 Km—L	5 to 10 Km—M	10 a 15 Km—N	15 to 20 Km—O	> 20 Km—P
**DAX**	0.14	0.96	1.95(0.22)	1.97(0.24)	1.95(0.23)	1.97(0.26)	1.97(0.22)
VAE	0.40	0.80	1.98(0.54)	1.98(0.56)	1.92(0.55)	2.02(0.55)	1.99(0.55)
PAE	0.21	0.92	1.23(0.21)	1.21(0.22)	1.21(0.24)	1.22(0.29)	1.23(0.23)
UVAE	0.25	0.90	1.60(0.41)	1.59(0.40)	1.62(0.40)	1.59(0.44)	1.64(0.38)
ACE	0.21	0.93	2.71(0.57)	2.78(0.55)	2.76(0.63)	2.76(0.53)	2.73(0.52)
**DAS**	0.98	0.41	2.92(0.74)	2.87(0.70)	2.85(0.69)	3.05(0.82)	2.95(0.72)
TO	0.87	0.47	2.95(0.80)	3.02(0.84)	2.97(0.79)	3.18(0.86)	3.05(0.83)
Dis	0.70	0.59	3.04(0.80)	2.93(0.76)	3.01(0.88)	3.12(0.89)	3.01(0.83)
ID	0.57	0.68	2.92(0.97)	2.88(0.93)	2.83(0.86)	3.03(0.95)	2.94(0.88)
SL	2.21	0.06	2.69(1.07)	2.44(0.93)	2.40(0.93)	2.71(1.20)	2.65(0.99)
**DDDI**	0.65	0.62	1.99(0.50)	1.92(0.43)	1.95(0.45)	2.00(0.56)	2.00(0.56)
AD	0.20	0.93	1.83(0.67)	1.82(0.63)	1.81(0.61)	1.90(0.81)	1.85(0.66)
NE	0.85	0.49	2.56(0.67)	2.41(0.62)	2.46(0.61)	2.49(0.76)	2.53(0.77)
RD	0.72	0.57	1.68(0.49)	1.61(0.39)	1.66(0.44)	1.70(0.51)	1.69(0.54)

For the gender based and driving associated to work (driving associated to work vs driving not associated to work) analyses, a t-test was made to compare the mean scores. From this test, it was possible to obtain the t-value (t, which is a measure of the relative difference between the two samples) and p-value (p, which is associated with the null hypothesis, being our null hypothesis that the groups that we are comparing are equal) [70]. In order to conclude that there is a significant difference between the mean scores of the two groups, the p-value must be less than 0.05 as we chose a 95% confidence level. For analyses based on age, approximate driving time and distance an ANOVA was conducted, obtaining an F-value (which is the ration of between-group variability and within-group variability) and a p-value for the possibility of differences between means. Also a Tukey Post Hoc test was conducted (when a significant difference was obtained in the ANOVA) having significant differences among groups when p ≤ 0.05. Before all the analyses were done, the assumptions of the statistical test were verified.

**Fig 3:**
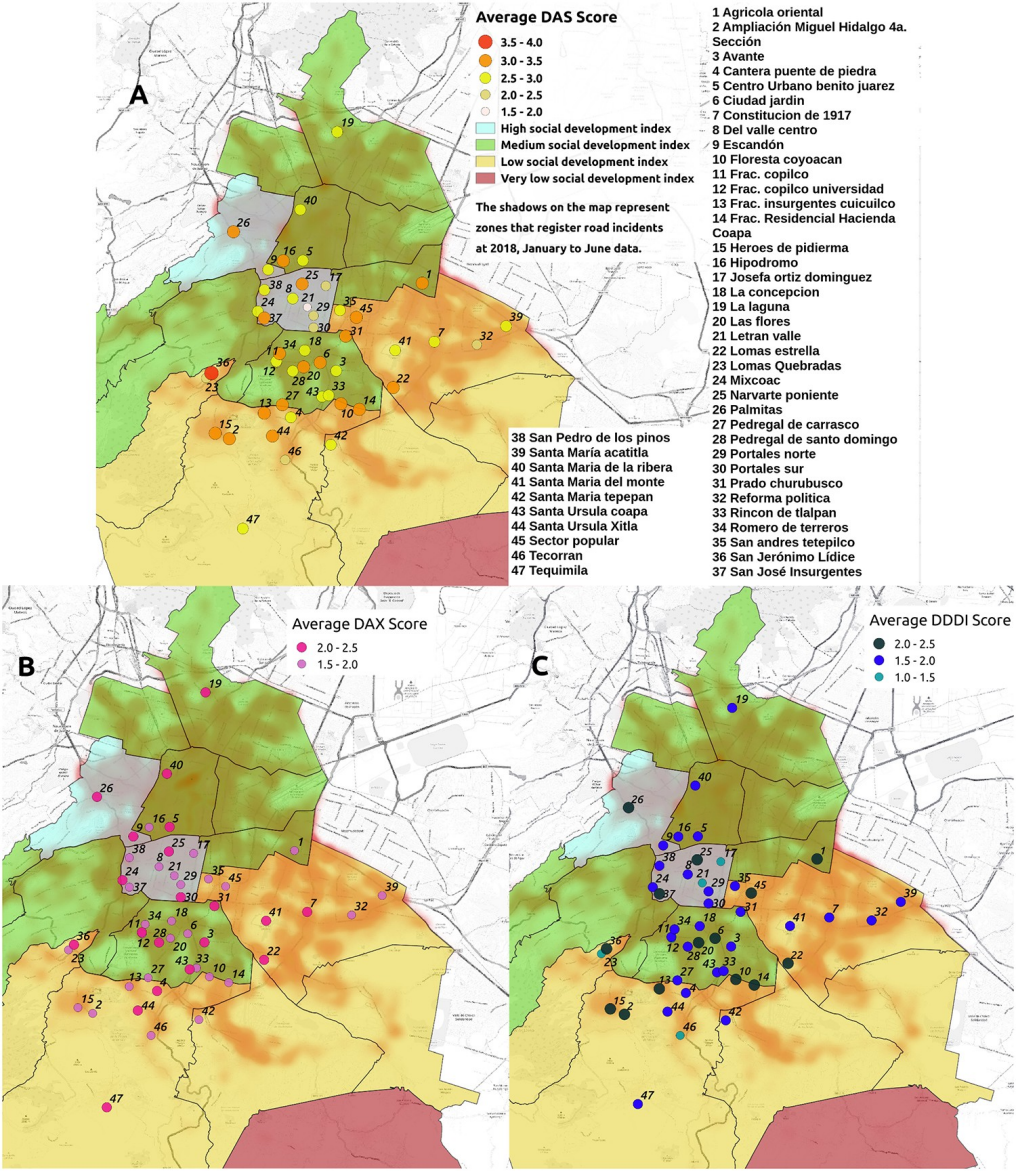
DAX, DAS and DDDI average scores on some neighborhoods in Mexico City. A. Total DAS average score. B. Total DAX average score. C. Total DDDI average score. The values are located on the map accordingly with the living place of at least three volunteers as they indicate in the geographical and personal information survey. These figures were done using the software QGIS (GPL license).

We graphed the DAX, DAS, and DDDI scores and location of each volunteer on a Mexico City map. This treatment of data was made with the QGIS software which has a GPL license for free software [71]. In this case, the average score obtained per neighborhood (obtaining an average of the score if there lived three or more volunteers) were represented as points on the map, and the value of the score for each survey was shown using a color and size code. Also, data related to the social development index of each municipality of Mexico City on 2015 [72] and road accidents and incidents in the City through January to June 2018 [73, 74] were included. The resulting visualization can be seen in Fig 3 in the next section.

## Results

### Geographic and demographic information

The total number of participants in the study was 626. From the information that volunteers registered on the platform, 48% of the participants were males and 52% were females. 21% were 18 to 29 years old, 32% were 30 to 39 years old, 29% were 40 to 49 years old, and 18% were 50 years or older. From the fourth question of the first questionnaire (see Supporting Information S1 File) we learnt how much time volunteers invested in driving. 52% of the volunteers drove 0.5 to 3 hours per day, 39% drove 3 to 6 hours per day, and 9% drove 6 hours or more. 1% of the participants had a secondary school degree, 10% had a high school degree, 50% had a bachelors degree, and 39% had a postgraduate degree. Also, from the 626 volunteers, 33% of them mentioned that their driving is associated with work, and 67% of the participants said that their driving is not associated with work. The whole sample was also divided accordingly with the distance from origin to destination. In this case, it was necessary to suppress from the sample volunteers that did not have a fixed working place or live and work in the same neighborhood. For the remaining 517 participants, we calculated the shortest paths between the locations and their distances. According to the calculations from these 517 participants, 17% of the volunteers travelled less than 5 Km from home to work, 33% travelled between 5 and 10 Km, 25% covered a distance between 10 and 15 Km, 12% travelled from 15 to 20 Km and 13% travelled a distance of 20 Km or more.

### General analysis

A first general analysis of the three surveys was made without considering any distinction between volunteers. A confidence test was made using the Cronbach’s *α* method. The *α* value is shown in Table 1 for every instrument and also every sub-scale. The literature considers adecuate a value of *α* over 0.7 [75] and in our case all *α* satisfied this condition except for the DAX subscale Use of Vehicle for Aggressive Expression (UVAE) (*α* = 0.66) and the DDDI subscale Omitted Items (*α* = 0.29). The analysis showed that in the case of the UVAE subscale, the only item with a negative correlation was “I follow right behind the other driver for a long time” and we decided to remove the question for the analyses (also we removed this question for the total DAX calculations). Then, we repeated the test and obtained a Cronbach’s *α* for the UVAE subscale over 0.7. Since the *α* value for the DDDI subscale Omitted Item was too low (*α* = 0.29), we decided not to take it into account for the analyses (including the total DDDI calculations). Fig 1 shows the histograms for every survey and a scatter graph which shows the mean score for each sub-scale. From Fig 1 it is possible to observe that for DAX and DDDI surveys, most of the volunteers show low expressions of anger. On the DAX, most of the volunteers show scores between 1 and 2.5 (Fig 1A), and for DDDI most of the drivers show scores between 1 to 3 (Fig 1B). However, for the DAS survey, which is useful to know the drivers’ anger level, most of the participants obtain scores closer to 3 and many show higher than four (Fig 1C). Fig 1D shows the mean scores for each sub-scale of each survey. The DAX score obtained in each case was multiplied by 1.25 to have 5 as a maximum value. Table 1 shows the average score obtained in Mexico City for every survey and each sub-scale.

### Analyses done for any characteristic

The next set of analyses were performed based on the participants characteristics, dividing the whole sample into groups. The first analysis was made based on volunteers’ gender and the resulting sample was: 302 males (group A) and 324 females (group B). The second analysis was based on the ages of the volunteers and were separated into four groups: 18 to 29 years (group C, 129 volunteers), 30 to 39 years (group D, 198 participants), 40 to 49 years (group E, 184 volunteers) and 50 or more years (group F, 115 volunteers). The third analysis was made taking into account the approximate driving time daily invest by volunteers. In this case, the sample was separated into three groups: 0.5 to 3 hours (group G, 328 participants), 3 to 6 hours (group H, 243 participants) and 6 or more hours (group I, 55 volunteers). The fourth analysis considered whether the volunteers driving was associated with work or not. The first group of volunteers mentioned that driving is part of their work (an assignment duty or because it was needed for them to move around the city) (group J, 204 volunteers). The second group responded that they do not drive because of work (group K, 422 participants). The last analysis was made in accordance with the origin-destination distance. In this case, 109 volunteers were removed from the sample, because they do not have a fixed working place or they lived and worked in the same neighborhood. In this last case, the reduced sample (517) was divided into five groups: participants who travel less than 5 Kilometers (group L, 86 participants), 5 to 10 Km (group M, 171 volunteers), 10 to 15 Km (group N, 129 participants), 15 to 20 Km (group O, 63 volunteers) and 20 or more Km (group P, 68 participants).

#### Analysis by gender

The results of the analysis based on gender are shown in Table 2. In this analysis, there are no significant differences in the total DAX and total DAS scores between males (group A) and females (group B). For the total DDDI score, there is a significant difference between the male and female mean score. On the DAX sub-scales, women show a significant difference in ACE with men, while men show a significant difference on UVAE compared to women. On VAE and PAE sub-scales, men and women do not show significant differences. For the DAS survey, females show a significant difference with men on the TO subscale. In the DDDI sub-scales, men show significant differences with women in AD and RD. From Fig 2A, it is possible to see the mean scores obtained for each group on every subcale.

#### Analysis by age

On Table 3, we show the significant differences between groups for every survey and every subscale based on age. For the DAX survey, volunteers in groups C and D show significant differences with groups E and F in the UVAE subscale. Groups C and D show significant differences with group F in VAE. Group D shows a significant difference with group F in the PAE and ACE sub-scales. Group C shows significant differences with groups E and F on the total DAX score. For the DAS survey, group C shows a significant difference with group E in the SL subscale. On the DDDI survey, group C shows a significant difference with groups E and F in the total DDDI score. Group D shows a significant difference with group F in AD too. The mean scores for every group on every subscale can be seen in Fig 2B.

#### Analysis by driving time

A third analysis was performed considering the approximate number of daily driving hours. For this analysis, an ANOVA test were performed (see Table 4 for results). Only group G shows a significant difference with group I in the PAE subscale, and this is the only significant difference (p≤0.05 on the Tukey post hoc test) found for this analysis. On Fig 2C it is possible to see the mean values for each group on every subscale.

#### Analysis by driving associated to work

For the fourth analysis, it is possible to observe in Table 5 that, for the DAX survey, volunteers in group J show a significant difference with group K in DAX sub-scales VAE, PAE, and UVAE. For the DAS survey, group J shows a significant difference with group K in TO, Dis, and SL sub-scales. Also, for the DDDI survey, group J shows a significant difference with group K in all sub-scales. On Fig 2D, it is possible to see all the scores obtained for each group on each subscale.

#### Analysis by traveling distance

A fifth analysis was made taking into account the distance between living place and working place for each volunteer. This analysis was made with a sample of 517 volunteers working in different neighborhoods and have a fixed working place. In this case, the sample was divided into five groups. Data was analyzed using an ANOVA. In this case, all groups showed similar values in all scales and sub-scales. Detailed values of ANOVA (F and p values) are show in Table 6. The mean score obtained for each group on every subscale are shown Fig 2E.

#### Data mapping in Mexico City

In Fig 3, it is possible to observe the average score by neighborhood for total DAX, DAS, and DDDI. The size of the point visually represents the score for each instrument (bigger the point, bigger the average of the total score). It can be seen, in Fig 3A, a significant concentration of big size points in the south-west part of the city. The neighborhoods in this zone are San Jeronimo Lídice, Ampliación Miguel Hidalgo 4a Sección, Héroes de Padierna, Santa Ursula Xitla, Fraccionamiento Insurgentes Cuicuilco, and Pedregal de Carrasco. The lowest average scores for DAS were obtained in Josefina Ortiz Dominguez, Letrán Valle, Portales Norte, and Portales Sur. In Fig 3B and 3C, the average score for DAX and DDDI total score are respectively represented. Also, the data related to the social development index (colored areas) and road accidents and incidents on the different zones (shadows in the map) were included.

## Discussion

**Fig 4:**
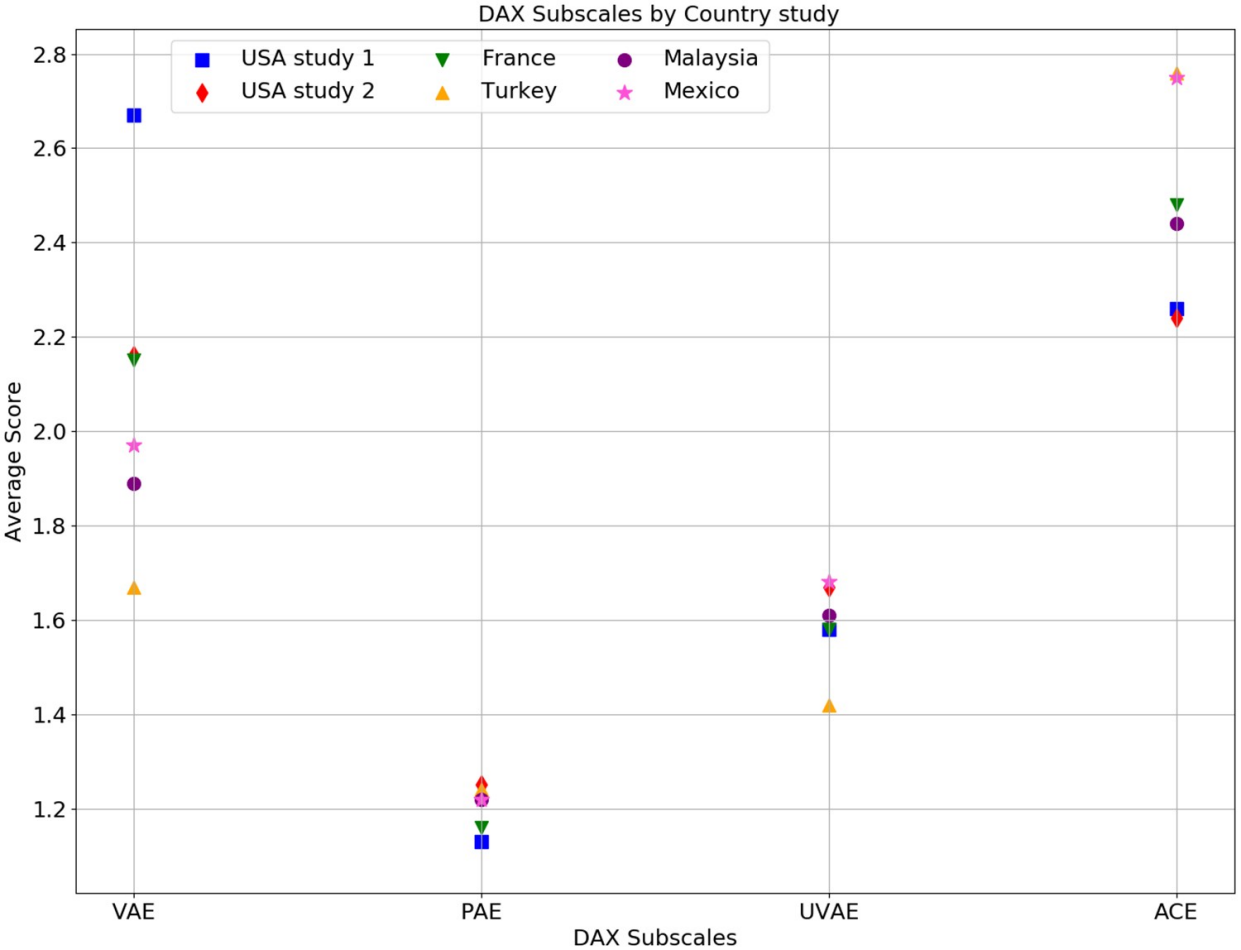
DAX sub-scales average value for different countries. The values obtained for all DAX sub-scales for two studies made for researchers in USA [32, 35], France [76], Turkey [46], Malaysia [36], and the one obtained in this work.

It is well known that Mexico City is one of the cities with the highest vehicular congestion in the world [60, 61]. For this reason, driving anger is an important topic for this particular population. The data obtained in this study allowed us to measure anger level and also the ways people express their anger while driving. Moreover, our results shown in Table 1, can be compared to the results from similar studies performed in other places (see Fig 4).

For the VAE sub-scale (DAX survey), people in Mexico City show a low score (1.97(0.56)) compared with the studies made in the USA (2.67(0.65) [32] and 2.16(0.55) [35]) and France (2.15(0.96)) [76]; and show a higher score compared with Turkey (1.67(0.39)) [46], and Malaysia (1.89(0.54)) [36]. On the results obtained for Physically Aggressive Expression sub-scale, Mexico City citizens showed a similar score as those in the mentioned studies. For sub-scale UVAE, participants in Mexico City show a higher score compared with participants in other countries (see Fig 4). For the last DAX sub-scale, ACE, Mexico City shows a higher score (2.75(0.59)) compared with the scores published by USA (2.26(0.84) and 2.24(0.55)), France (2.48(0.94)), and Malaysia (2.44(0.61)). See Fig 4.

As for the DAS survey, Mexico City drivers show a higher score than Chinese drivers (professional and non-professional) [29] in all sub-scales. More results can be compared for this scale. However, it is essential to acknowledge that the survey in other studies contains more items [19, 21–24, 77]. Indeed, a small comparison has been made between those results. For example, Mexico City citizens show a lower score on the TO sub-scale (3.04(0.84)) compared with the scores obtained in the USA (3.30) [19] and Malaysia (3.30) [77]. Also, in Mexico City, the score is higher in the same sub-scale compared to China (2.70) [21], Japan (2.10) [24] and Spain (2.77(0.74)) [22]. Also for the ID sub-scale, Mexican City drivers show a lower score (2.91(0.93)) compared with Spain (3.46(0.88)) and Turkey (3.50(0.88)). However, in Mexico City, the score in this sub-scale is higher than those in USA (2.70), China (2.54), Japan (2.20), and Malaysia (2.75(0.95)).

Comparing our results obtained by gender analysis with the ones published by Dula and Ballard (DDDI survey) [30], Mexico City inhabitants score lower. In the particular case of males, in Mexico City the total score (as the sum of each item score) is 63.03(16.75) while the one shown by Dula is 70.73(11.79) [30]. For females in Mexico City, the total score is 59.38(13.76) while the value obtained in [30] is 65.68(13.74). For the rest of sub-scales, all the scores obtained for Mexico City are lower.

From all the analysis we made, there is a set of findings we believe is relevant to discuss, the first one is gender differences, the second one is age, a third one related with driving as part of work, and a final one focused on the geographical anger hotspots in the city. For gender, in the DAX survey, females show a higher score and a significant difference with males in ACE. On the UVAE sub-scale, males showed a higher score than females (and also a significant difference). Our results seem consistent with some other studies. For example, Deffenbacher, White and Lynch [52] found that men showed a greater vehicular expression on the 49-items DAX survey. Deffenbacher and colleagues’ study found that males show a small Adaptive/Constructive behavior compared to women, nonetheless, there was no difference between genders in verbal expression of anger (university sample, 436 students from the United States). In a related study, Deffenbacher et al. [78] found that men report a high UVAE score and females report a high ACE score (university sample, 372 students from the United States). Also, Eşiyok, Yasak, and Korkusuz found that females from Ankara, Istanbul, and Samsun express their anger in an Adaptive/Constructive way [51]. Jovanović et al. also found that women from Serbia report more Adaptive/Constructive behaviors [50]. In this study, for the DAS survey, women showed a higher score and a significant difference with men in TO and no difference in the total DAS score. These results are different from the ones reported in other studies. For example, Sullman found that females from New Zeland show a higher score in the total DAS score (using the 33-items DAS survey) [48]. In another work, Sullman et al. [22] found that females from Girona, Spain report more anger for TO, ID and Dis sub-scales.

For the DDDI survey, in this study, men show a higher score and a significant difference with women on AD and RD sub-scales. Also, Dula and Ballard [30] found that males living in North Carolina, in the United States showed a higher score than females in AD and RD sub-scales. All this information suggests that even when women are more reactive to situations that could cause anger (like in our case in the TO DAS sub-scale), it seems that females try to express their anger in a more constructive and adaptive way compared with males. This is similar to the tendencies we observe. The existence of anger differences among genders has been studied not only for driving. For example, Fernández and Malley-Morrison [79] conducted a critical review of the prevalence and expression of anger in males and females. Some studies do not show gender differences [80, 81]. However, Fernández and Scott [82] report differences in intensity (higher for males), frequency and duration of anger (higher for females). Concerning anger expression, the study of Archer [83] suggests a difference in the way males and females in the type of aggression that males and females adopt to express their anger.

Henessy et al. [84] assess different explanations for gender differences in anger expression and argue that males and females show no differences in mild forms of driving aggression like verbal aggression and aggression expressed by gestures. This similarity between males and females may be attributed, according to the authors to the anonymity of the driver and a small time of interaction between drivers. However, in situations that require prolonged contact, the sensation of anonymity is lost and then a gender difference is noticeable (males show more aggression and females prefer not to get involved on risky or dangerous situations), however this doesn’t explain the differences. What we believe is interesting to notice is the fact that to some point seems to be present everywhere regardless the gender role assumed locally. We do not subscribe ourselves to a biosocial hypothesis, but we wonder if there is something in driving in cities that affords such differences.

From the analysis made by age, on the DAX survey, the group F (50 years or older) show significant differences with other age groups in VAE, PAE, and UVAE. Also, group C and D have a significant difference with groups E and F in UVAE. Similar results were found for Herrero-Fernández [53]. In this work, he found that younger drivers report a higher score in the aggressive expression sub-scales. On the DAS survey, participants with ages between 18 to 29 years old show a significant difference with the group of participants with ages between 40 and 49 in SL. A similar result was also found for Jovanović et al. [50]. They found that older and experienced drivers report less anger and also less aggressive behavior while driving. On the DDDI survey, volunteers in group D showed a significant difference in AD sub-scale with group F. A possible explanation for the results obtained for the age analysis, is that older participants probably prefer to adopt behaviors that let them calm down, controlled their anger more, and think less of revenge. On the other hand, young drivers prefer to express externally their anger and also prefer to let the others know what they feel or think. These assumptions can explain why older adults show the lowest levels of anger and the highest score in ACE sub-scale and younger drivers show the highest levels of anger and also the highest scores in DAX sub-scales like VAE and UVAE. The studies made by Møller and Haustein [85], and Phillips et al. [86] give some support to this explanation. Møller and Haustein found in their study that young people often want to show others that they made a mistake. Phillips et al. found that older adults show with less frequency their anger externally and have less probability to say disgusting comments or argue. Also, Phillips et al. found that older adults report with more frequency the use of strategies that let them preserve their calmness and also think less in the actions done by others against them [86].

Also, differences were found for anger associated with driving as part of work. For the VAE, PAE, and UVAE sub-scales in the DAX survey, the group of participants that drive more for work requirements shows the highest scores, compared with the group of volunteers whose driving is not associated to work. Also, the first group shows the highest score in all DAS sub-scales and DDDI sub-scales. In this case, it seems that the ones who have to drive more because of work feel more anger and also express it more. In the work of Feng et al. [3] professional drivers show the lowest score in TO, Dis and SL sub-scales and the highest score in ID sub-scale. For the Mexico City case, people that drive more due to work requirements are not necessarily professional drivers or the ones that spend more time behind the wheels. For future work, a study on public transportation drivers, goods transportation drivers or any person who require a professional license to work would be interesting.

Finally, results over a Mexico City map are shown in Fig 3. It is possible to see that the lowest scores (the average of at least three participants that live in the same neighborhood) are in the downtown of the city while some of the highest are located in some neighborhoods of the south-west zone of the city. In Fig 3 shows that differences in scores are not related to the social development index (SDI). Low SDI is present in the east (which shows low driving anger scores) and south-west zones of the city (which shows higher driving anger scores). Also, some of the lowest driving anger scores are located in zones where road incidents and accidents are frequently reported (darker shadows). One possible explanation is related to geographical and road conditions. For example, roads in downtown are wide and mostly flat. In contrast, in neighborhoods like Santa Ursula Xitla, roads are narrow and are located in a mountainous area. We believe that more research is needed in understanding the possible role of the geographical and topographical conditions in driving anger as it makes the driving experience more or less stressful.

We are aware of the descriptive nature of our work, however, in the future and based on the results presented here, we are interested in deepening in the causal-mechanistic aspects of the phenomenon and offer a more qualitative interpretation of it. In this line of thought, we will study the factors that could influence the distribution of anger in the city based on complex networks theory and data analysis (Fig 3).

The initial aim of this work was to observe if there are some tendencies in the level and the expression of anger from participants with different characteristics. This information would be useful to include these differences in agent-based models on traffic games. In recent years, computational modeling of social issues has been useful to understand the complexity of multidisciplinary problematics and have been taken force as simulations and modeling are good tools for prediction. The inclusion of agents characteristics and behaviors are helpful to improve previous models [8] and study emergence phenomena due to driving anger.

### Limitations of the study

This study has some limitations that are important to mention. First of all, only drivers who have access to the internet were able to participate. Also, even when an effort was made to obtain equitable participation of Mexico city drivers, the sample was not distributed homogeneously over the city. Another limitation is that it was not enough participants that drive for a long time or work as professional drivers. It is important to take into account that the assessments were all based on questionnaires rather than actual behavior or self-monitored behavior. Therefore, the findings may be distorted by recall difficulties and social desirability bias. Also, it is important to take into account that the surveys were applied online and many of the previous studies were applied using pen and paper. This difference in the acquisition of data can impact the results due to different factors as the recruitment methods, study samples and survey administration. It is important to consider the differences between the instruments used in this study and the instruments used in previous research work. In general, the work gives a wide vision of the driving anger phenomena and let a guideline for future and more detailed work.

## Conclusion

In general, citizens of Mexico City show differences on DAS and DAX sub-scale scores with drivers in other countries. Also, Mexico City drivers show a higher score in the Adaptive/Constructive Expression on the DAX survey. Another finding is that women show high score in Adaptive/Constructive Expression, while men show a high score in Use of Vehicle for Aggressive Expression. On DAS, females show to be more reactive to traffic obstruction than males. Also, participants with ages between 18 and 29 show significant differences with the volunteers of ages up to 50, with younger drivers showing a high level of anger and also a high expression of that anger.

## Supporting information

S1 File Personal and geographic information survey. This survey is related to personal information as sex, age, hours of driving and geographic information as living and working places.
(PDF)
