# Supplementary material for: Anger while driving in Mexico City
Source: PLoS One. 2019 Sep 30;14(9):e0223048. doi: 10.1371/journal.pone.0223048 (PMC6768538; doi:10.1371/journal.pone.0223048)
Supplement: S1 File — This survey is related to personal information as sex, age, hours of driving and geographic information as living and working places. (PDF) [file pone.0223048.s001.pdf]

Personal and Geographical Information Survey

This survey was made to know necessary information about volunteers as well geographic information and driving habits.

1. Please, indicate your age in years (open space to write)

2. Please, indicate your gender __male __ female

3. Do you drive for more time due to work requirements of any kind? __yes __no

4. How many hours do you drive daily approximately? (open space to write)

5. At which hours do you drive often? __ 6 to 9 am __ 9 to 12 pm __12 to 3 pm __3 to 6 pm __6 to 9 pm __ 9 to 12 am __12 to 3 am __3 to 6 am.

6. Have you been involved in car crashes or accidents? __yes __no

    * How many? (open space to write)

7. Do you live permanently in Mexico City? __no __yes

    * In which city do you live? (open space to write)

8. Please, indicate you last obtained degree __secondary school __ high school __bachelor __postgraduate

9. Do you work or study at UNAM? __yes __no

10. In which neighborhood do you live? (open space to write)

11. In which neighborhood do you work? (open space to write)

12. Do you think that men are better drivers than women? __yes __no

13. When you argue with another driver, is it more common that you have it with a man or a woman? __man _woman
